# Carbon Nanoparticles Inhibit Α-Glucosidase Activity and Induce a Hypoglycemic Effect in Diabetic Mice

**DOI:** 10.3390/molecules24183257

**Published:** 2019-09-06

**Authors:** Taili Shao, Pingchuan Yuan, Lei Zhu, Honggang Xu, Xichen Li, Shuguang He, Ping Li, Guodong Wang, Kaoshan Chen

**Affiliations:** 1 Anhui Provincial Engineering Research Center for Polysaccharide Drugs, Wuhu 241002, China; 2 Drug Research & Development Center, School of Pharmacy, Wannan Medical College, Wuhu 241002, China; 3 Anhui Province Key Laboratory of Active Biological Macromolecules, Wuhu 241002, China

**Keywords:** carbon nanoparticles, polysaccharide, *Arctium lappa* L. root, hypoglycemia, diabetes mellitus

## Abstract

New, improved therapies to reduce blood glucose are required for treating diabetes mellitus (DM). Here, we investigated the use of a new nanomaterial candidate for DM treatment, carbon nanoparticles (CNPs). CNPs were prepared by carbonization using a polysaccharide from *Arctium lappa* L. root as the carbon source. The chemical structure and morphology of the CNPs were characterized using Fourier-transform infrared spectroscopy, X-ray photoelectron spectroscopy, elemental analysis, and transmission electron microscopy. CNPs were spherical, 10-20 nm in size, consisting of C, H, O, and N, and featuring various functional groups, including C=O, C=C, C–O, and C–N. In vitro, the as-prepared CNPs could inhibit α-glucosidase with an IC_50_ value of 0.5677 mg/mL, which is close to that of the reference drug acarbose. Moreover, in vivo hypoglycemic assays revealed that the CNPs significantly reduced fasting blood-glucose levels in mice with diabetes induced by high-fat diet and streptozocin, lowering blood glucose after intragastric administration for 42 days. To the best of our knowledge, this is the first report of CNPs exhibiting α-glucosidase inhibition and a hypoglycemic effect in diabetic mice. These findings suggest the therapeutic potential of CNPs for diabetes.

## 1. Introduction

Diabetes mellitus (DM) is one of the most serious chronic endocrine disorders in the human body worldwide, and according to statistics, more than 400 million global populations have been afflicted with it up until 2017, making it currently the most prevalent metabolic disease. The number of those suffering from diabetes may presumably increase dramatically to about 640 million in 2045 [1]. DM is usually classified as either insulin dependent (type 1) or insulin independent (type 2), and 90% of diabetes patients are diagnosed with type 2 DM. In type 2 DM, cells in the body become insulin resistant leading to perturbations in the insulin signaling pathway and impaired glucose absorption in tissues such as adipose and muscle, which is characterized by high blood glucose [2,3]. In order to lower blood glucose, one therapeutic approach for mitigating postprandial hyperglycemia is to retard the absorption and digestion of carbohydrate molecules in the gastrointestinal tract via inhibition of a key enzyme α-glucosidase [4,5]. α-Glucosidase plays an important role in the lysis of α-glucopyranoside bonds in oligosaccharides and disaccharides to release monosaccharides that are subsequently absorbed into the body, regulating glucose availability and the degree of postprandial hyperglycemia. Thus, the inhibition of α-glucopyranoside is considered an important target for the discovery and development of new more potent, less toxic anti-diabetic drugs. Since 1990, three α-glucosidase inhibitor compounds, acarbose, voglibose, and miglitol, have been used clinically to enhance the biological processes involved in the reduction of glucose absorption [6,7,8,9,10]. However, while their efficacy in inducing a hypoglycemic effect has been proven, these drugs can also aggravate gastrointestinal symptoms, including nausea, bloating, diarrhea, abdominal pain, and flatulence [11]. Therefore, the development of novel therapeutic drugs for diabetes is currently an urgent need.

Carbon nanoparticles (CNPs) have garnered significant interest from researchers in the field of nanometric scale materials. CNPs can be made in a straightforward manner, using various carbon sources to produce a variety of different functional groups on their surfaces. Therefore, they may be easily derived and functionalized to obtain the particular properties required for specific applications. For example, CNPs with good solubility, intense fluorescence, high resistance to photo-bleaching, low toxicity, or good biocompatibility may be prepared. On account of their unique properties, CNPs have potential value for applications in bioimaging, biosensing, and drug delivery. Moreover, a variety of different functionalized CNPs have been developed, and they are increasingly being used as antibacterial and anticancer agents, expanding the development of CNPs applications in the medical field [12,13,14,15]. Recently, Sun et al. reported that carbon dots derived from a source selected because of its use in traditional Chinese medicine showed a hypoglycemic effect in a mouse model in which hyperglycemia had been induced by intraperitoneal injection of glucose [16]. However, thus far, there have been no reports of CNPs inducing hypoglycemia in diabetes models. A polysaccharide obtained from the *Arctium lappa* L. root has been demonstrated to exhibit hypoglycemic activity, but it suffers from poor water solubility, which limits its applicability because of the associated low bioavailability levels [17,18,19,20,21]. In order to improve its water solubility, we focused our investigations on structural modifications of polysaccharides. In this study, we synthesized CNPs via hydrothermal treatment of polysaccharides obtained from *Arctium lappa* L. root and studied their inhibition activity toward α-glucosidase in vitro as well as their hypoglycemic effect in vivo on mice with diabetes induced by high-fat diet and streptozocin (STZ) treatment.

## 2. Results and Discussion

The accepted preparation procedure for CNPs is carbonization, which includes hydrolysis, dehydration, decomposition, condensation, aromatization, and passivation. During these steps, aromatic centers are formed, and eventually, carbon cores are created [22,23,24,25]. In order to obtain CNPs with maximized bioactivity, the impacts of temperature, concentration, and reaction time were studied. Based the preliminary investigations, the temperature was critical for the preparation of CNPs. It was observed that the inhibition of α-glucosidase activity was the most obvious when the CNPs were prepared at the temperature of 180 °C. When the temperature was below 180 °C, the procedure of carbonization would not happen thoroughly. The inhibition of α-glucosidase activity was reduced by increasing the temperature, up to 220 °C, the activity disappeared due to the carbon source polysaccharides from fresh *Arctium lappa* L. root was decomposed completely (Figure S1, Supporting Information). On the above results, the CNPs could not be synthesized even if the temperature is too low or high. Further, upon increasing the reaction time up to 2 h, the CNP bioactivity increased, and the latter also increased with concentration. Herein, 180 °C, 2 h, and 0.01 g/mL were chosen as the optimum conditions for the synthesis of the CNPs.

Next, absorption and fluorescence spectra of the CNPs were acquired. As shown in Supporting Information Figure S2, the UV-vis absorption spectra of the CNPs exhibit absorption peaks between 200 and 300 nm, which could possibly be assigned to n-σ* transitions in OH and NH groups and n-π* transitions in C=O, N=O, N=N, and C=N groups on the surface of CNPs. As the excitation wavelength was increased from 360 to 450 nm, the CNPs emission gradually shifted to longer wavelengths (Supporting Information Figure S3). This excitation-dependent fluorescence behavior is one of the most characteristic properties of CNPs. The absolute quantum yield at 480 nm was measured to be 12% using the calibrated integrating sphere.

**Figure 1:**
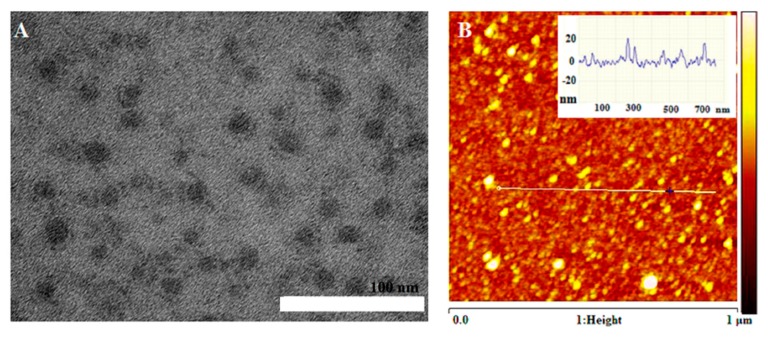
(**A**) Transmission electron microscopy and (**B**) Atom force microscopic images of the as-prepared CNPs.

**Figure 2:**
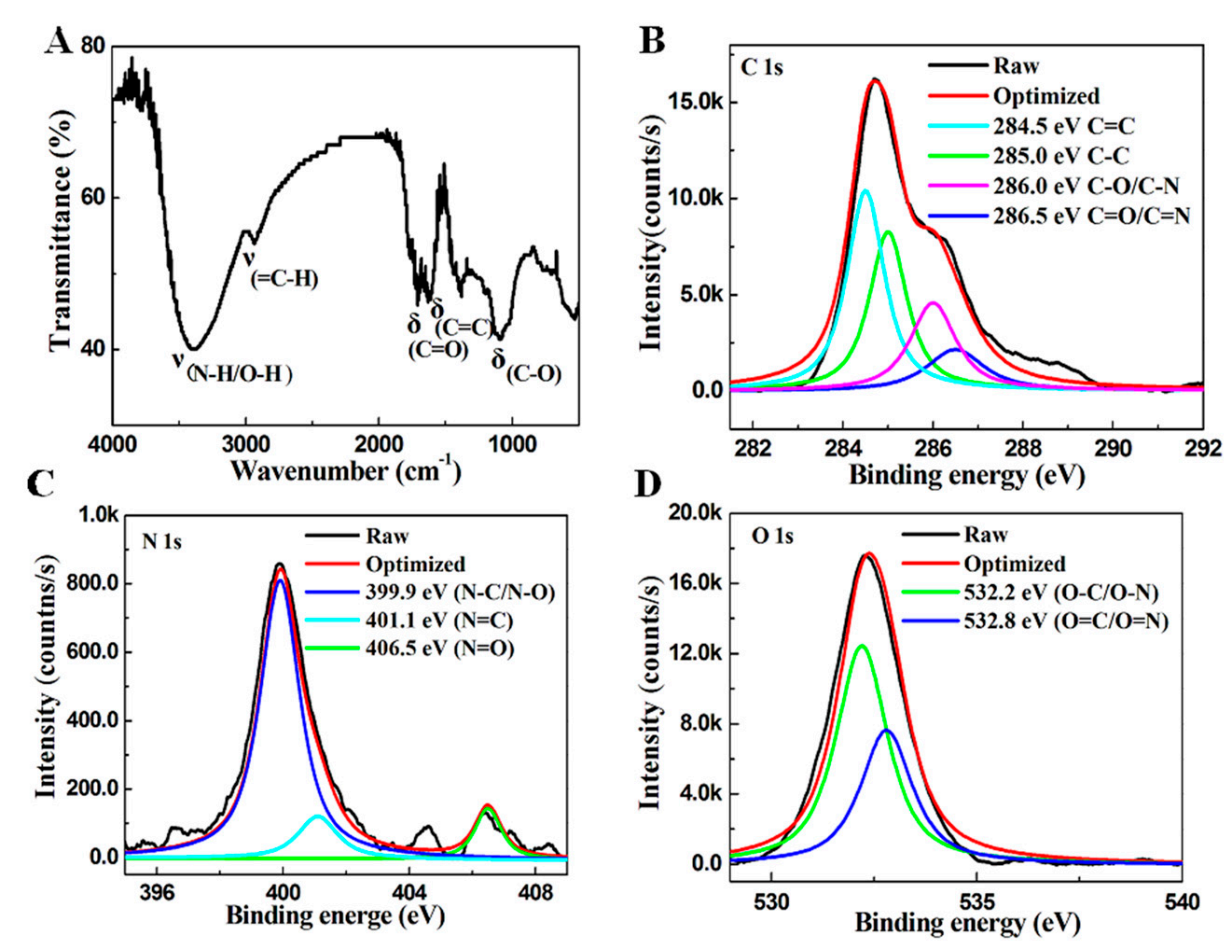
Spectra of the as-prepared CNPs. (**A**) Fourier-transform infrared spectrum; (**B**) C 1s, (**C**) N 1s, and (**D**) O 1s X-ray photoelectron spectra.

The morphology of the CNPs was examined by means of transmission electron microscopy (TEM) and atomic force microscopic (AFM). As shown in Figure 1A, the TEM image reveals that the CNPs are well dispersed, with an estimated size of 10–20 nm. Their height is 10–20 nm, based on AFM measurement (Figure 1B). Therefore, the as-prepared CNPs are quasi-spherical. Functional groups in the CNPs were distinguished by Fourier-transform infrared (FT-IR) spectroscopy (Figure 2A). The FT-IR spectrum exhibits distinct absorption bands at 3400, 1725, 1610, 1350, and 1250 cm^−1^, corresponding to NH/OH, C=O, C=C, C–N, and C–O groups, respectively. For a better understanding of the composition of the CNPs, Elemental analysis (EA) and X-ray photoelectron spectroscopy (XPS) measurements were performed. The EA results revealed that the CNPs contained the four elements C, O, N, and H, at amounts of 41.73, 49.58, 2.08, and 6.25%, respectively. The C 1s XPS spectrum (Figure 2B) can be decomposed into five peaks, centered at 284.5, 285.0, 286.0, and 286.5 eV and assigned to C=C (sp^2^), C–C (sp^3^), C–O/C–N, and C=N/C=O, respectively. The appearance of a peak at a binding energy of 284.4 eV indicates the presence of graphitic sp^2^ carbon structures. The N 1s spectrum (Figure 2C) can be resolved into peaks at 399.9, 401.1, and 406.5 eV, attributed respectively to C–N/N–O, C=N, and N=O. The O 1s spectrum (Figure 2D) consists of three peaks, at 532.2, 532.8, and 533.8 eV, which are assigned to C–O/N–O, C=O/N=O, and C=O, respectively [26,27,28].

**Figure 3:**
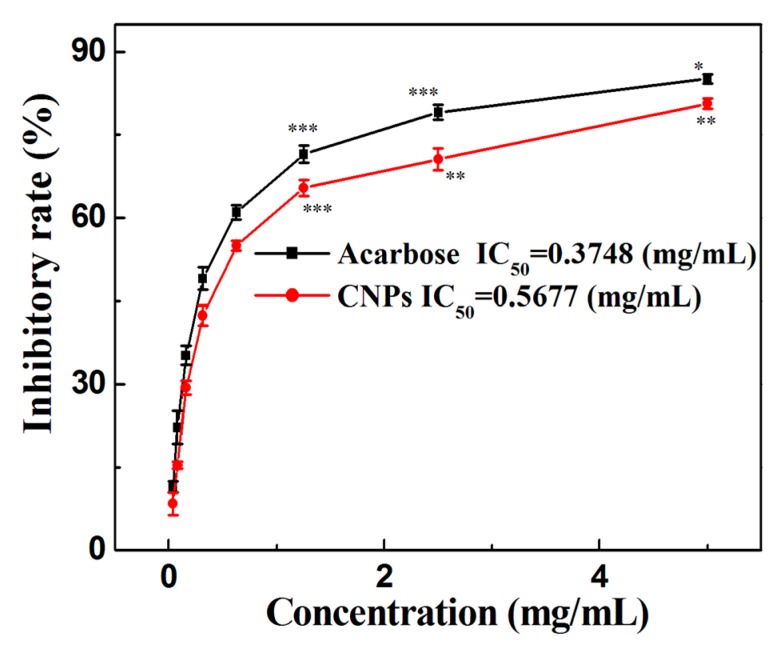
Inhibitory effects of the as-prepared CNPs and acarbose on α-glucosidase activity. Data ware expressed as mean ± standard deviation (*n* = 3), * *p* < 0.05, ** *p* < 0.01, *** *p* < 0.001 vs. blank control.

Figure 3 presents the results of the α-glucosidase inhibitory activity assay for the as-prepared CNPs. α-glucosidase inhibitors (e.g., acarbose) are drugs that can aid glycemic control in diabetes, slowing the post-prandial rise in blood glucose, and improving the body’s use of insulin [11]. The as-prepared CNPs exhibit a potent α-glucosidase inhibitory efficacy with an IC_50_ value of 0.5677 mg/mL. This effect is close to that observed for acarbose, the reference drug. A possible enzyme inhibition mechanism for the CNPs is as follows: the CNPs could combine with α-glucosidase by noncovalent bonding to alter the structure of the enzyme. They may occupy the active sites or other sites on α-glucosidase, and then, the α-glucosidase/CNP hybrids are likely to hinder the combination of the enzyme with the substrate, reducing enzymatic activity. Another possibility is that the α-glucosidase/CNP hybrids combine with substrate molecules to form an α-glucosidase/CNP/glucose complex that is not capable of releasing the enzymatic lysis products, and hence a lower blood-glucose level is produced [29].

**Figure 4:**
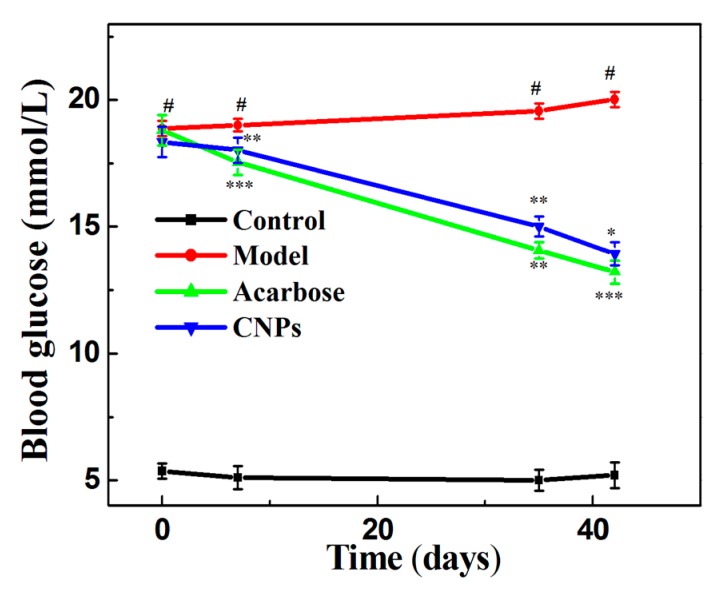
Effects of as-prepared CNPs and acarbose on blood-glucose levels in mice. Data ware expressed as mean ± standard deviation (*n* = 8), ^#^ *p* < 0.001 vs. control, * *p* < 0.05, ** *p* < 0.01, *** *p* < 0.001 vs. model.

**Figure 5:**
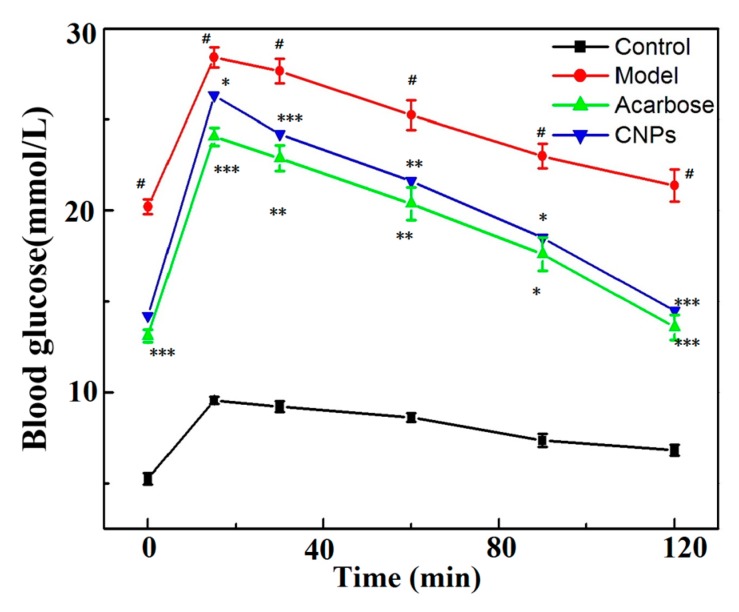
Effects of the as-prepared CNPs and acarbose on glucose tolerance in mice. Each value is expressed as mean ± standard deviation (*n* = 8), ^#^ *p* < 0.001 vs. control, * *p* < 0.05, ** *p* < 0.01, *** *p* < 0.001 vs. model.

Figure 4 illustrates the effect of the experimental treatments on blood-glucose levels in the four experimental groups. In this work, the normal control group (healthy mice) had blood-glucose levels in the range of 4.85-5.04 mmol/L during the course of the study. The model group (untreated diabetic mice) showed a progressive increase in blood glucose (*p* < 0.001), produced by the toxic effect of STZ. In contrast, the acarbose and CNPs groups (diabetic mice received reference drug acarbose and CNPs, respectively) had a progressive reduction in blood glucose. The fasting blood-glucose levels in the CNPs group were higher than those in the acarbose group, but they were lower than those in the model group after 42 days (*p* < 0.01, *p* < 0.001). CNPs may compete with carbohydrates in the bloodstream, reducing their bonding with α-glucosidase, resulting in a potent α-glucosidase inhibitor [7]. As shown in Figure 5, blood-glucose levels in mice from each group reach their highest level 15 min after the oral administration of glucose aqueous solution (40%), the levels fasting blood-glucose of CNPs group were lower than those in the model group remarkably (*p* < 0.01). The result indicated the CNPs significantly improved the glucose tolerance in diabetic mice. This finding suggests the hypoglycemic potential and feasibility of using CNPs as a therapeutic approach. This point is further supported the low cytotoxicity of as-prepared CNPs. During the test, the oral, ocular, and nasal membranes of CNPs group (diabetic mice that received 200 mg/kg of the CNPs daily during the assay) preserved a normal aspect. No changes in the skin and coat were detected, and the somatomotor activity and behavior were normal. The experiment finished with 100% survival. This evidence suggests that CNPs did not produce toxic effects on experimental animals when was administered in a dose of 200 mg/kg.

## 3. Materials and Methods

**Scheme 1:**
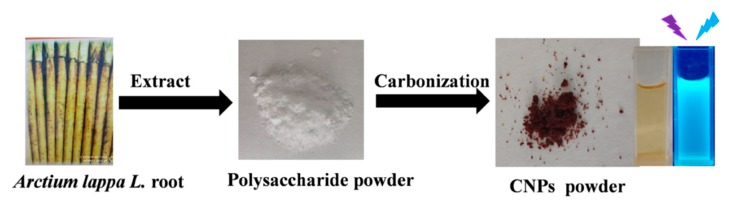
Graphical representation of the synthesis route for the CNPs.

A white powder consisting of polysaccharides was obtained from fresh *Arctium lappa* L. root via hot-water extraction, alcohol precipitation, decolorization, deproteinization, and freeze-drying, according to a reported method [18,19,30,31,32]. CNPs were fabricated from the obtained polysaccharide by one-step hydrothermal carbonization. Typically, 2 g of polysaccharide was dissolved in 20 mL of Milli-Q water, transferred into a 50 mL Teflon-lined stainless-steel autoclave, and then incubated at 180 °C for 2 h. The resulting dark brown solution was cooled to room temperature and then centrifuged at 10000 rpm for 10 min. The supernatant was filtered through a 0.22 μm filter membrane to further remove large particles and aggregates. Finally, the CNPs solution was dialyzed using another membrane (cut-off: 500–1000 Da) against Milli-Q water for 48 h, and then lyophilized for further use [26]. The synthesis route is depicted in Scheme 1.

The ultraviolet-visible (UV-vis) absorption spectrum of the prepared CNPs were measured using a UV-vis spectrophotometer (UV-7000, GE, Boston, MA, USA) with 1 cm matched quartz cuvettes using the wavelength range from 200 to 400 nm. The fluorescence measurements were performed on spectrofluorophotometer (F-4600, Shimadzu, Kyoto, Japan) equipped with a R3896 red-sensitive multiplier and a 1 cm quartz cuvette upon excitation at 360–450 nm with a slit width of 10 nm. The morphology and size of the CNPs were characterized by transmission electron microscopy (TEM) (Tecnai G2 20 ST, FEI, Portland, OR, USA,) operating under the accelerating voltage of 200 kV, a drop of CNPs dispersed with distilled water were dropped onto a carbon-coated copper grid and then left to dry under ambient conditions. Atom force microscopic (AFM) topography images were acquired from an Innova scanning probe microscope, a drop of CNPs dispersed with distilled water were dropped onto a mica and then left to dry under ambient conditions. CNPs were freeze-dried for further composition analysis. Surface chemical bonding states were analyzed by X-ray photoelectron spectroscopy (XPS) (ESCALAB250, Thermo Scientific, Boston, MA, USA). Fourier-transform infrared (FT-IR) measurements were carried out with a FTIR spectrometer (FTIS-8400S, Shimadzu) using a KBr plate, the measurement conditions were as follow: the scan range of 500–4000 cm^−1^, 500 scans, resolution 4 cm^−1^. Elemental analysis (EA) of the CNPs was executed using a Vario MACRO cube (Elemetar Analysensysteme GmbH, Hanao, Germany). Using a fluorescence spectrometer (PLS920, Edinburgh Photonics, Edinburgh, UK), the absolute quantum yields, *η*, of the CNPs were determined and are defined as follows:

(1) $$\eta \;=\frac{\varepsilon }{\alpha }=\frac{\int L_{\mathrm{emission}}}{\int E_{\mathrm{solvent}}-\int E_{\mathrm{sample}}}$$

where *ε* and *α* represent the proportion of photons emitted and absorbed, respectively, by the sample. The luminescence emission spectrum *L*_emission_ and the excitation spectra of the specimen *E*_sample_ and solvent *E*_solvent_ were obtained with the aid of a calibrated integrating sphere (Edinburgh Photonics PLS920, Edinburgh, UK).

To test the CNPs in vitro, the glucosidase inhibitory activity was determined according to the method of Prada et al., with slight modifications, by measuring the release of 4-nitrophenol from 4-nitrophenyl-α-d-glucopyranoside (4-NPGP) [7]. A 5 mM solution of 4-NPGP in 0.1 M sodium phosphate buffer (pH 6.8) was prepared. To each well of a microplate, a 20-μL aliquot of the acarbose, control (DMSO), or CNPs solution was added, along with 20 μL of the enzymatic solution (1 U/mL). The microplate was incubated for 10 min at 37 °C, in the dark. Next, 20 μL of 4-NPGP was added to each well, followed by incubation for a further 30 min. Finally, 100 μL of a Na_2_CO_3_ solution (0.2 M) was added to terminate the reaction. Absorption at 405 nm was measured using a microplate reader. The analysis was performed in triplicate, and the percent inhibition (%*I*) was calculated according to Equation (2) and compared with the control group:

(2) $$\%I=100-\left[A_{a/p}-A_{c}\right]\;\times 100\%$$

where *A*_a/p_ is the absorbance of the sample minus the absorbance of the blank and *A*_c_ is the absorbance of the negative control minus the absorbance of the blank. Samples with inhibition ≥ 50% were used for the estimation of the IC_50_ concentration value, which was determined by Probit analysis using GraphPad Software (GraphPad Software 4.0), at a level of significance of 0.05 [7,33].

In vivo, SPF C57BL/6 black male mice (18 ± 2 g), supplied by Nanjing Qinglongshan Animal Breeding Farm (Nanjing, China), were used. All animal experiments were approved by the Ethics Committee for the Use of Laboratory Animals, Wannan Medical College. First, the animals were acclimatized in specific-pathogen-free laboratory for a week, in a temperature-controlled environment at 23 ± 2 °C, a relative humidity of 60 ± 10%, and with a 12/12 h light–dark cycle. Diabetes was then induced by injection of STZ (50 mg/kg, every 24 h) after administering high-fat diet for 4 weeks; seven administrations of STZ were completed, high-fat diet feeding-caused diabetes in mice is generally accompanied by early symptoms of dyslipidemia, whose prominent features include elevated TG, TC, and LDL-C levels, and decreased HDL-C levels, playing a key role in the development of the pathogenesis of diabetes [34,35,36]. At the end of this period, the blood-glucose levels in fasted mice were determined using a standard glucometer. Animals with blood-glucose measurements higher than 16.5 mmol/L were included in the subsequent assay experiments. The animals were randomized into four groups (eight animals in each group):

- Normal control group: Healthy mice that received a normal diet throughout the assay.
- Model group: Diabetic mice that were not treated. They received distilled water throughout the assay.
- Acarbose group: Diabetic mice that received 200 mg/kg of the reference drug daily during the assay [7,37].
- CNPs group: Diabetic mice that received 200 mg/kg of the CNPs daily during the assay.

All treatments were administered orally, using an intragastric cannula. The animals were given free access to food and water throughout the assay experiment. Blood-glucose levels were determined in blood samples taken from the tail vein on days 0, 7, 35, and 42 using a standard glucometer.

To test the oral glucose tolerance, the mice were fasted for 14 h after the 42-day administration period, and their blood glucose was measured in blood samples taken from the tail vein. Then, the mice were orally treated with 2.0 g/kg of 40% glucose aqueous solution, and their blood glucose levels were measured at 15 min, 30 min, 60 min, 90 min and 120 min.

SPSS 13.0 software was used for data analysis. Means and standard deviations were obtained for each result. Comparisons between pairs of groups were made using a *t*-test for unpaired samples. To compare more than two groups, a one-way ANOVA was carried out, followed by Tukey’s HSD test. Differences were considered to be statistically significant at *p* < 0.05.

## 4. Conclusions

CNPs with an estimated size of 10 to 20 nm were prepared and demonstrated to produce an obvious hypoglycemic effect when administered to mice with diabetes induced by high-fat diet and STZ. We also showed that the mechanism of action of this effect probably involves inhibition of α-glucosidase activity. As far as we know, this is the first article reporting an in vivo hypoglycemic effect of CNPs derived from *Arctium lappa* L. root polysaccharides. This study was a preliminary evaluation of the hypoglycemic bioactivity of the CNPs. Further investigations are required in order to elucidate not only the exact mechanism but also the structure-activity relationships underlying this effect.

## Supplementary Materials

The following are available online at https://www.mdpi.com/1420-3049/24/18/3257/s1, Figure S1: Effect of temperature on the α-glucosidase activity. The concentration of as-prepared CNPs is 5 mg/mL. Figure S2: UV-vis absorption spectrum of the as-prepared CNPs dispersed in water. Figure S3: Fluorescence spectra of the as-prepared CNPs in water at different excitation wavelengths.
