# Supplementary material for: Carbon Nanoparticles Inhibit Α-Glucosidase Activity and Induce a Hypoglycemic Effect in Diabetic Mice
Source: Molecules. 2019 Sep 6;24(18):3257. doi: 10.3390/molecules24183257 (PMC6767295; doi:10.3390/molecules24183257)
Supplement: Supplementary file 1 [file molecules-24-03257-s001.pdf]

Carbon Nanoparticles Inhibit A-Glucosidase Activity and Induce a Hypoglycemic Effect in Diabetic Mice

Taili Shao [1,2], Pingchuan Yuan [1,2,3], Lei Zhu [1,2,3], Honggang Xu [2], Xichen Li [2], Shuguang He [1,2], Ping Li [1,2], Guodong Wang [1,2,3,*] and Kaoshan Chen [1,2,3,*]

1 Anhui Provincial Engineering Research Center for Polysaccharide Drugs, Wuhu 241002, China
2 Drug Research & Development Center, School of Pharmacy, Wannan Medical College, Wuhu 241002, China
3 Anhui Province Key Laboratory of Active Biological Macromolecules, Wuhu 241002, China
* Correspondence: guodong201@csu.edu.cn (G.W.); ksc313@126.com (K.C.); Tel.: +86-553-3932414 (G.W.); +86-553-3932489 (K.C.)

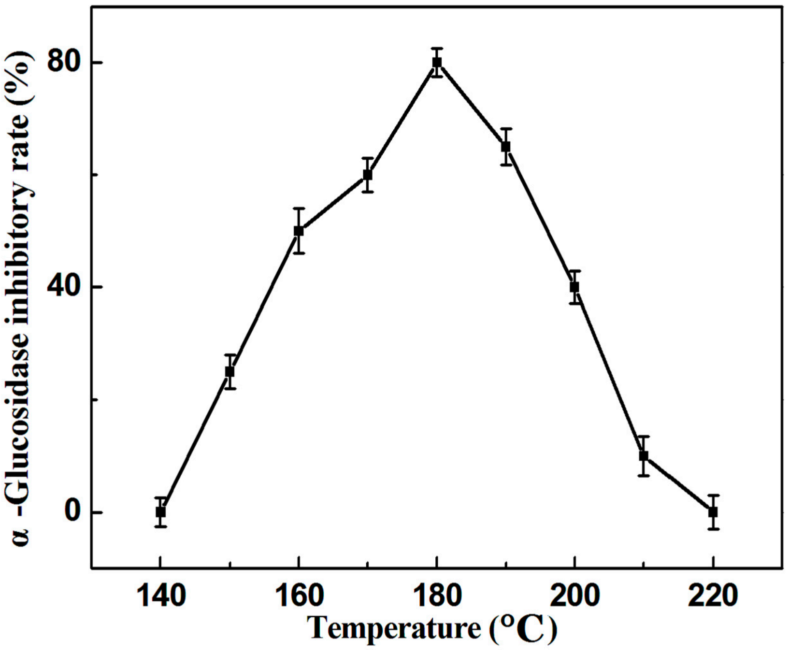

Figure S1. Effect of temperature on the α-glucosidase activity. The concentration of as-prepared CNPs is 5 mg/mL

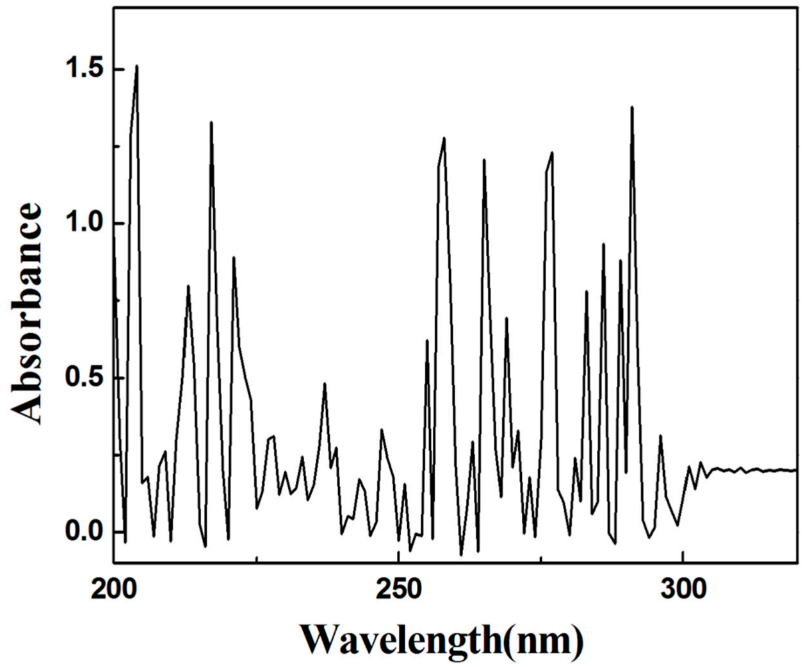

Figure S2. UV-vis absorption spectrum of the as-prepared CNPs dispersed in water.

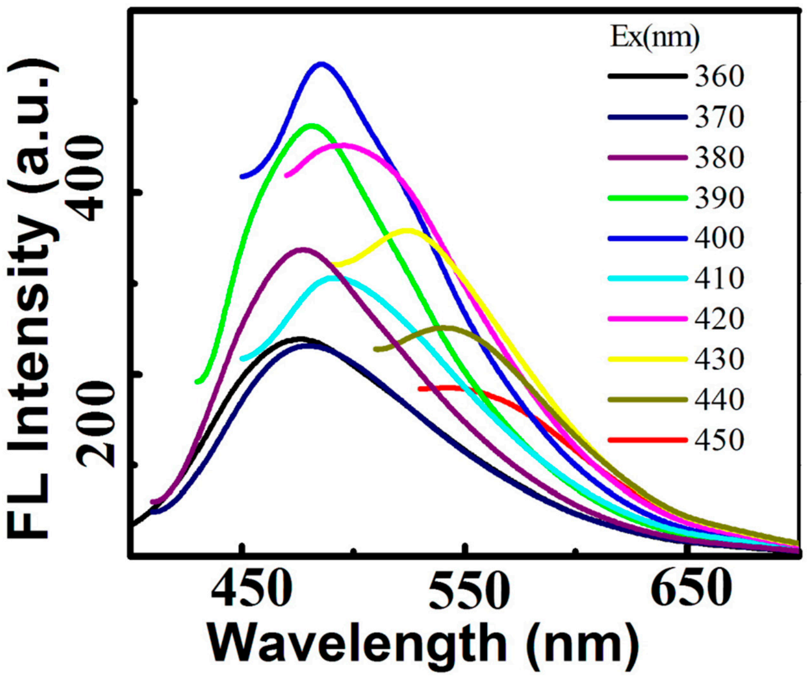

Figure S3. Fluorescence spectra of the as-prepared CNPs in water at different excitation wavelengths.
